# Syntaxin-17-Dependent Mitochondrial Dynamics Is Essential for Protection against Oxidative-Stress-Induced Apoptosis

**DOI:** 10.3390/antiox8110522

**Published:** 2019-10-30

**Authors:** Binran Wang, Xiaoyue Xiao, Fanwei Huang, Rong Liu

**Affiliations:** 1 College of Food Science and Technology, Nanjing Agricultural University, Nanjing 210095, China; u201513068@hust.edu.cn (B.W.); xxy10458@foxmail.com (X.X.); u201512875@hust.edu.cn (F.H.); 2 Department of Pathogen Biology, School of Basic Medicine, Huazhong University of Science and Technology, Wuhan 430030, China; 3 National Center for International Research on Animal Gut Nutrition, Nanjing 210095, China; 4 Jiangsu Collaborative Innovation Center of Meat Production and Processing, Nanjing 210095, China

**Keywords:** lysosome, autophagy, membrane fusion, mitochondrial fission, ROS, oxidative stress

## Abstract

In this study, cell death induced by the oxidant tert-butylhydroperoxide (tBH) was observed in U_2_OS cells; this phenotype was rescued by Syntaxin 17 (STX17) knockout (KO) but the mechanism is unknown. STX17 plays dual roles in autophagosome–lysosome fusion and mitochondrial fission. However, the contribution of the two functions of STX17 to apoptosis has not been extensively studied. Here, we sought to dissect the dual roles of STX17 in oxidative-stress-induced apoptosis by taking advantage of STX17 knockout cells and an autophagosome–lysosome fusion defective mutant of STX17. We generated STX17 knockout U_2_OS cells using the clustered regularly interspaced short palindromic repeats (CRISPR)/CRISPR-associated protein 9 (Cas9) system and the STX17 knockout cells were reconstituted with wild-type STX17 and its autophagosome–lysosome fusion defective mutant. Autophagy was assessed by autophagic flux assay, Monomer red fluorescent protein (mRFP)–GFP–LC3 assay and protease protection assay. Golgi, endoplasmic reticulum (ER)/ER–Golgi intermediate compartment (ERGIC) and mitochondrial dynamics were examined by staining the different indicator proteins. Apoptosis was evaluated by caspase cleavage assay. The general reactive oxygen species (ROS) were detected by flow cytometry. In STX17 complete knockout cells, sealed autophagosomes were efficiently formed but their fusion with lysosomes was less defective. The fusion defect was rescued by wild-type STX17 but not the autophagosome–lysosome fusion defective mutant. No obvious defects in Golgi, ERGIC or ER dynamics were observed. Mitochondria were significantly elongated, supporting a role of STX17 in mitochondria fission and the elongation caused by STX17 KO was reversed by the autophagosome–lysosome fusion defective mutant. The clearance of protein aggregation was compromised, correlating with the autophagy defect but not with mitochondrial dynamics. This study revealed a mixed role of STX17 in autophagy, mitochondrial dynamics and oxidative stress response. STX17 knockout cells were highly resistant to oxidative stress, largely due to the function of STX17 in mitochondrial fission rather than autophagy.

## 1. Introduction

Excessive reactive oxygen species (ROS) produced both exogenously and endogenously leads to DNA damage, cell structure damage and ultimate cell death. Exogenous ROS can be triggered by stimuli such as tBH and H_2_O_2_, while endogenous ROS primarily derives from mitochondria. At least two strategies are used by cells to remove these ROS and avoid cell death caused by ROS induced irreversible damages. The first strategy is antioxidant enzymes such as superoxide dismutase (SOD), catalase and glutathione peroxidases (GSH-Px or GPx). In addition, mitophagy is another strategy to scavenge endogenous ROS through elimination damaged mitochondria which release ROS into cytoplasm. So far, two types of mitophagy—PINK1/Parkin dependent and independent—have been identified and well-studied. Fragmentation of mitochondria is the first step for both types of mitophagy. ROS stimuli induce mitochondria fragmentation and then proceed to mitophagy. While elongated mitochondria escape from autophagic degradation and maintains adenosine triphosphate (ATP) production to be partially protected from apoptosis [1]. Therefore, reactive oxidants induce mitophagy, while the induced mitophagy removes the ROS source reversely to protect cells from cell death. However, overload ROS cause acute fragmentation of mitochondria and cytochrome C release which activate caspases and substrates cleavage. Ultimately irreversible programmed cell death, such as apoptosis or necrosis, is triggered [2,3].

Syntaxin 17 (STX17) is proposed to have a function at the ER–mitochondria contact sites where it promotes mitochondrial fission by determining dynamin-related protein 1 (Drp1) localization and activity [4]. Starvation disrupts the STX17–Drp1 interaction and thus favors mitochondrial elongation during autophagy. 

STX17 also plays a critical role in autophagosomal fusion with lysosomes [5,6]. STX17 localizes to the outer membrane of completed autophagosomes via its C-terminal hairpin structure mediated by two tandem transmembrane domains containing glycine zipper-like motifs, where it interacts with synaptosome associated protein 29 (SNAP29) and the lysosomal SNARE vesicle associated membrane protein 8(VAMP8) to assemble into a four-α-helix bundle [5]. By “zippering” the α-helix bundle, the trans-SNARE autophagic complex can bring the membranes of autophagosomes and lysosomes into close apposition and induce membrane fusion by reducing the hydration repulsive barrier. As in many fusion processes, autophagic SNAREs are regulated by SNARE-associated proteins and membrane tethering factors, including autophagy-related protein 14 (ATG14) in the oligomeric state, which physically interacts with STX17 and SNAP29 and possesses membrane tethering activity [7] and the homotypic fusion and protein sorting complex that also interacts with STX17 [8,9]. 

Emerging evidence suggests that the function of STX17 is not limited to mature autophagosomes. STX17 was also found to cycle between the endoplasmic reticulum (ER), ER–Golgi intermediate compartment (ERGIC) and Golgi [10]. STX17 also localizes to the ER–mitochondria contact sites where it recruits ATG14 and activates autophagosome biogenesis [11]. 

The divergent functions of STX17 in autophagosome biogenesis, autophagosomal fusion with lysosomes, mitochondrial fission and ER–Golgi maintenance remains to be validated and differentiated in mammalian cells. Since essentially most of the loss-of-function experiments on STX17 have been performed by RNA interference, the incomplete elimination of STX17 may impede the justification of STX17 being required in divergent cellular processes. We generated STX17 null U_2_OS cell lines using clustered regularly interspaced short palindromic repeats (CRISPR)/CRISPR-associated protein 9 (Cas9) targeted genome editing and reconstituted STX17 knockout (KO) cells with a autophagy defective form of STX17 to dissect the functions of STX17 in autophagy and mitochondrial dynamics during oxidative stress response. Here, we confirmed STX17 is essential for autophagosome-lysosome fusion and aggrephagy and completed depletion of STX17 negatively regulates mitochondria elongation and mitochondrial ROS production. Autophagy defective form GFP-STX17 caused severest cell death and mitochondrial ROS production by rescuing both tBH induced mitochondria fragmentation and inhibiting of autophagosome-lysosome fusion. While STX17-Flag, the full function form of STX17, shows minor effects due to rescue both of mitochondria fragmentation and autophagosome-lysosome fusion. This finding dissects the dual role of STX17 in ROS induced cell death and autophagy.

## 2. Materials and Methods

### 2.1. Reagents and Antibodies 

Mouse anti-Flag (F3165), rabbit anti-STX17 (HPA001204, Sigma, Jacksonville Registrant State, USA), rabbit anti-LC3 (L7543), rabbit anti-ERGIC53 (MFCD07370310) and mouse anti-β-tubulin (T8328) antibodies were purchased from Sigma-Aldrich (Jacksonville, FL, USA). Mouse anti-LAMP2 (sc-18822) and mouse anti-Tom20(sc-17764) antibodies were purchased from Santa Cruz. Rabbit anti-p62 (PM045, MBL) antibody was purchased from MEDICAL & BIOLOGICAL LABORATORIES CO. (MBL) (Woburn, MA, USA). Mouse anti-p62 (H00008878-M01) antibody was purchased from Novus. Mouse anti-GM130 (610822) was purchased from BD Biosciences (San Jose, CA, USA). Mouse anti- conjugated ubiquitin FK2 antibody (PW8810) was purchased from Biomol Res. The antibodies are diluted according to the instructions of each company. 

### 2.2. Cell Culture, Cell Transfection and Cell Lysate Preparation

U_2_OS cells were cultured in dulbecco’s modified eagle medium (DMEM) (Sigma) supplemented with 10% FBS (HyClone) and 1% Penicillin–Streptomycin Solution (Gibco). Cell transfection was performed using Lipofectin 2000 (Invitrogen) or PEI (Polysciences) according to the protocols provided by the manufacturers. Cell lysates were mixed with sodium dodecyl sulfate (SDS) loading buffer (200 mMTris-HCl, pH 6.8, 400 mM dithiothreitol (DTT), 16% β-mercaptoethanol, 8% SDS, 23 loading dye base (Amresco) and 40% glycerol), boiled for 10 min and subjected to SDS–polyacrylamide gel electrophoresis (PAGE) and Western blotting according to standard protocols.

### 2.3. Immunofluorescence Staining 

Cells were transfected according to the protocol described above with noted plasmids. Twenty-four hours after transfection, cells were trypsinized and transferred to six-well dishes containing coverslips. After another 24 h, cells were fixed using ice-cold methanol for 4 min at 4 °C. Cells were then washed three times with phosphate buffered saline (PBS) and blocked with blocking buffer (2.5% BSA 1 0.1% Triton X-100 in PBS) at 25 °C for 2 h. Cells were incubated with primary antibodies at 4 °C overnight, washed with PBS buffer and then incubated with appropriate secondary antibodies for 2 h at 25 °C. Slides were examined using a laser scanning confocal microscope (Zeiss LSM 510 META ultraviolet–visible). To monitor the ΔΨm, cells were treated with 150 nM MitoTracker Orange CM-H2-TMRos or 50 nM tetramethylrhodamine ethyl ester perchlorate (TMRE) for 30 min. Cells were washed two to three times and subjected to live-cell imaging for TMRE staining. mRFP-GFP-LC3 stable cell line was generated in U2OS cells. The cells were treated with Rapamycin for acidification assay. 

### 2.4. Construction of Stable Knockout Cell Lines Using the CRISPR/Cas9 Technique

Scanning GN19NGG motifs on the genome was used to identify anti-STX17 guide RNA candidates that fit with the rules for U6 Pol III transcription and the protospacer adjacent motif (PAM) recognition domain of spCas9. To avoid off-target cleavage of the human genome, the anti-STX17 guide RNA candidates were filtered to remove the high sequence homology within the entire human genome, especially removing the targets with fewer than three single-nucleotide mismatches or with low-fidelity sequences in positions 1–5 directly for 50 of the requisite NGG PAM of the CRISPR. The top five off-target candidates were picked from the CRISPR design program (http://crispr.mit.edu) and we cloned the flanking B500 bp from the genomic DNA of anti-STX17 CRISPR/Cas9 stable cells for TA cloning and DNA sequencing. The identification and verification of gene knockout events were based on both sequencing analysis of genome polymerase chain reaction (PCR) fragments of targeting loci and immunoblotting analysis using antibodies specifically targeting STX17 (Sigma, Jacksonville, FL, USA).

### 2.5. Statistical Analysis

Results were quantified by counting punctum formation and the statistical significance of differences between mean values (*p* < 0.05) was evaluated using the unpaired Student *t* test. At least three different visual fields containing at least 100 cells were counted for each condition. All data were expressed as the mean ± standard deviation. Immunofluorescent images of cells stained with a Golgi, ER or ER–Golgi intermediate compartment (ERGIC) marker were measured either by manual demarcation of the Golgi with a limiting polygon or by calculation of its area using ImageJ (National Institutes of Health, San Jose, CA, USA).

## 3. Results

### 3.1. Generation of STX17 Knockout U_2_OS Cells

**Figure 1:**
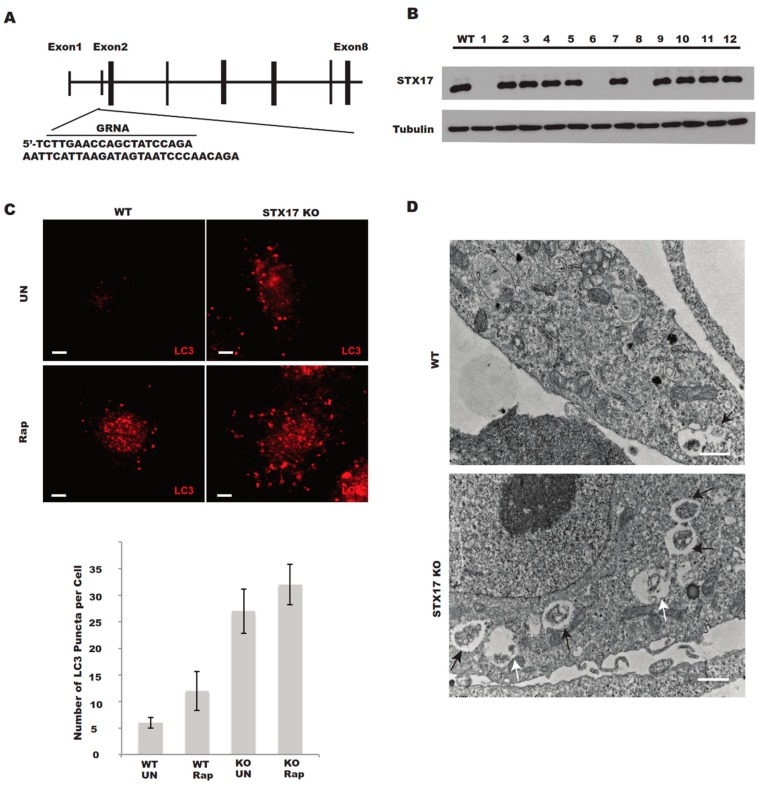
Generation of Syntaxin 17 knockout (STX17 KO) U_2_OS cells. (**A**) Knockout strategy for STX17 by the clustered regularly interspaced short palindromic repeats (CRISPR)/CRISPR-associated protein 9 (Cas9) system. The sequence within exon 1 targeted by single guide RNA is underlined. (**B**) Immunoblotting analysis of wild-type (WT) U_2_OS and 12 puromycin-resistant clones for STX17 knockout. (**C**) Upper panel—Immunostaining of endogenous LC3 in WT and STX17 KO U_2_OS cells. UN, untreated. Rap, rapamycin. Lower panel—Quantification analysis of the upper panel. Scale bar: 5 μm. (**D**) Electron microscopy analysis of WT and STX17 KO U_2_OS cells. Black arrows indicate autophagosomes. White arrows indicate autolysosomes. Scale bar: 2 μm.

The human osteosarcoma U_2_OS cell line is frequently used in the study of autophagy due to its relatively normal autophagic response [7,12,13]. We designed a small guide RNA (sgRNA) targeting the sequence within exon 2 of human STX17 (Figure 1A), which should lead to the complete destruction of STX17 genomic DNA and no expression of the STX17 protein. Indeed, we obtained 7 out of 24 clones with no STX17 protein expression and the STX17 expression of 12 clones including 3 STX17 null clones was shown via Western blotting analysis (Figure 1B). Since STX17 was completely knocked out in four of the knockout clones, we picked clone KO1 for the following functional analysis. We examined the autophagosome formation and accumulation in the STX17 knockout (KO) cells. LC3 puncta shown by immunostaining were significantly increased in STX17 KO cells (Figure 1C) and the majority of the autophagic membrane structures were sealed double-membrane autophagosomes with undigested cellular contents (Figure 1D). However, single-membrane autolysosome vesicles could also be observed in STX17 KO cell lines (Figure 1D). This suggests the accumulation of autophagic vacuoles in the STX17 KO cells.

### 3.2. Autophagosome–Lysosome Fusion is Partially Defective in STX17 KO Cells 

**Figure 2:**
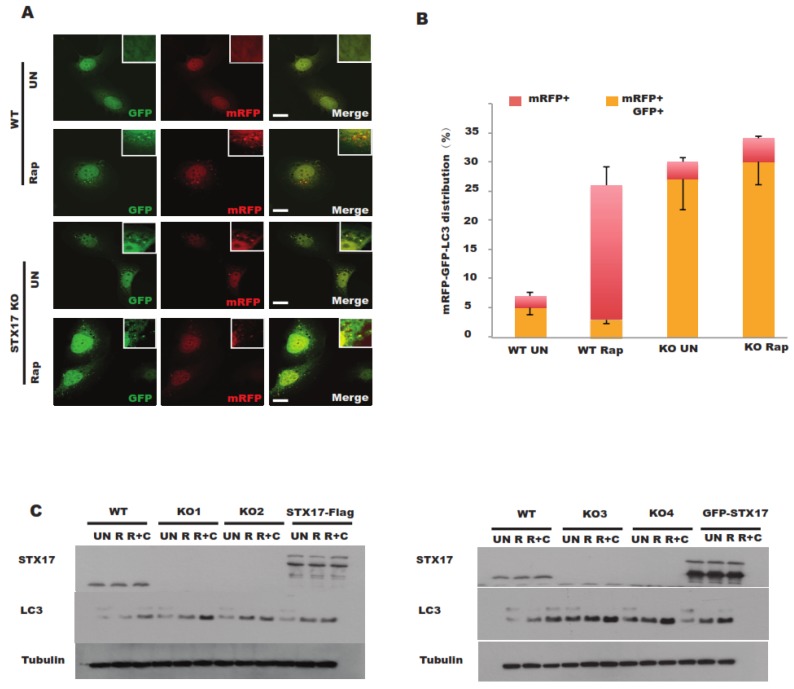
Autophagy flux is partially blocked in STX17 knockout U_2_OS cells. (**A**) WT and STX17 KO U2OS cells were transfected with monomer red fluorescent protein (mRFP)–GFP–LC3B. After 24 h, cells were treated with rapamycin before fixation and analyzed by confocal microscopy. Scale bar: 15 μm. (**B**) Quantitative analysis of acidified autophagosomes (mRFP + GFP−) versus neutral autophagosomes (mRFP + GFP+). (**C**) Analysis of the autophagy flux in four STX17 knockout clones upon different treatments for immunoblotting analysis. R, Rapamycin. CQ, Chloroquine. RC, Rapamycin + Chloroquine.

Once autophagosome–lysosome fusion is suppressed, the acidification of autophagosomes will be compromised [14]. We sought to examine the acidification of autophagosomes in STX17 knockout cells. Monomer red fluorescent protein (mRFP)-GFP-LC3 is a good marker to evaluate the acidification of autophagosomes since GFP is more sensitive to the acidic environment but RFP is resistant [7]. In STX17 KO cells, acidic autophagosomes (RFP+ only) were significantly reduced compared to in wild-type cells (Figure 2A,B), suggesting that the acidification of autophagosomes is compromised without STX17. 

Further, the autophagy flux was also examined in STX17 knockout clones. Consistent with a previous study using an RNA interference (RNAi) approach [5], STX17 was found to be required for autophagosome–lysosome fusion, characterized by greater accumulation of LC3-II in both treatment with or without Rapamycin (a mTOR inhibitor) in STX17 KO cells (Figure 2C) but LC3-II was further increased under chloroquine (CQ) treatment (Figure 2C). All four STX17 knockout clones presented a similar autophagy flux phenotype, albeit with slight differences among different clones, probably due to clonal variation. Flag-STX17 only partially rescued this phenotype, while GFP-STX17 did not (Figure 2C) due to the sterical effect of the GFP tag [15]. These data suggest that STX17 is utilized for autophagosome–lysosome fusion but is not essential, which is in line with previous studies [15]. 

**Figure 3:**
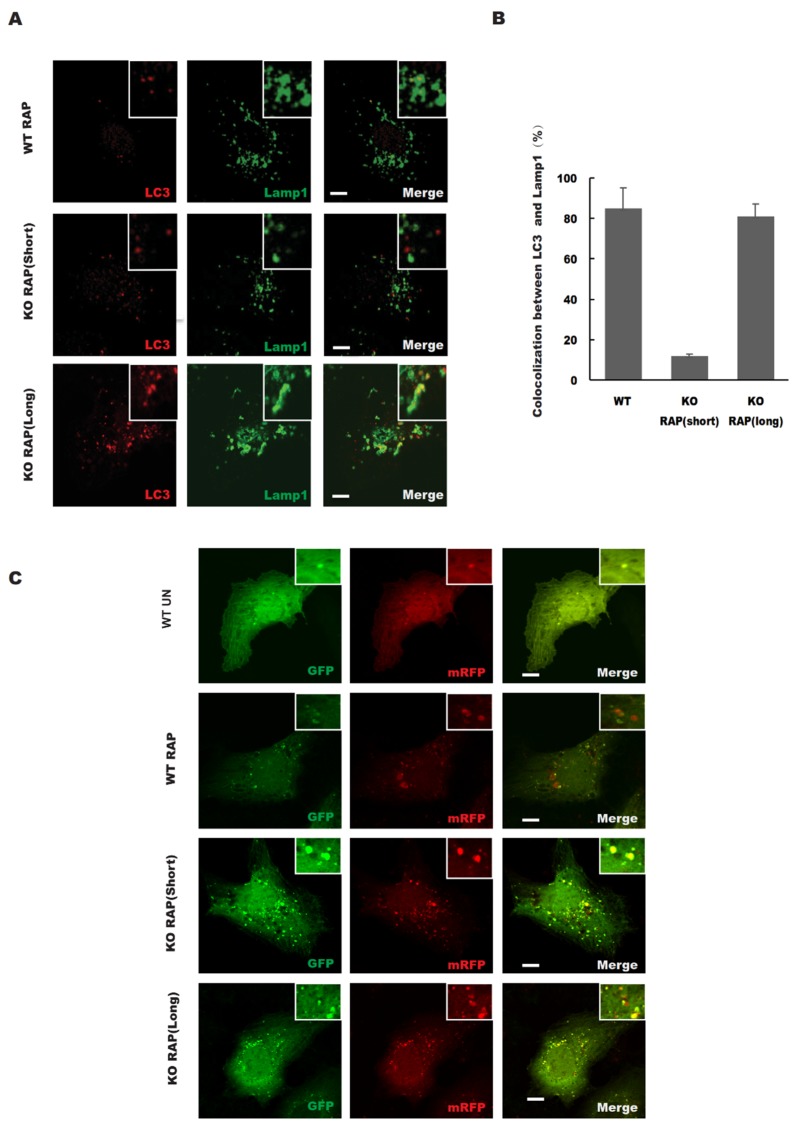
STX17 is required for autophagosome–lysosome fusion. (**A**) The colocalization between LC3 and lysosomal marker LAMP1 upon rapamycin treatment for different durations. Scale bar: 5 μm. (**B**) Quantitative analysis of A. (**C**) WT and STX17 KO U2OS stable expression mRFP–GFP–LC3B cells were treated with Rapamycin for different durations. Scale bar: 5 μm.

Consistent with the mRFP-GFP-LC3 data (Figure 3C), the colocalization between LC3 (autophagosomes) and LAMP1 (lysosomes) was significantly reduced in STX17 knockout cells (Figure 3A). However, we found that prolonged rapamycin treatment increased the colocalization of LC3 with LAMP1 in both wild-type and STX17 KO cells but with no significant difference (Figure 3A,B). Therefore, the data above suggest that autophagosome fusion with lysosomes is partially defective without STX17. 

### 3.3. The Role of STX17 in Organelle Dynamics

**Figure 4:**
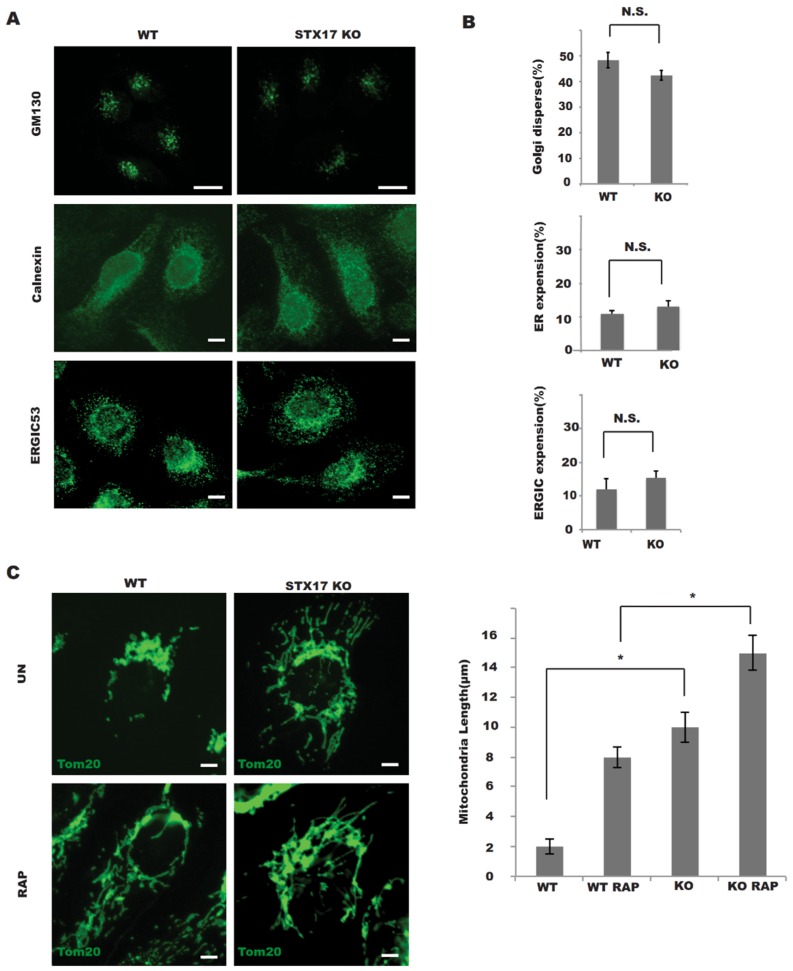
STX17 is required for mitochondrial fission but not for endoplasmic reticulum (ER)/ER–Golgi intermediate compartment (ERGIC)/Golgi architecture maintenance. (**A**) The morphology of the Golgi, ER and ERGIC marked by GM130, Calnexin and ERGIC53 staining was not altered in STX17 KO cells (Golgi dispersal was quantified by measuring the Golgi area per cell). Scale bar: 5 μm. (**B**) Statistics analysis of data presented in A. No Significance (N.S.) * Significance (**C**) Mitochondria were elongated in STX17 KO cells. Wild Type(WT) or STX17 KO cells were fixed and stained with Tom20 endogenous antibody after Rapamycin treatment. Scale bar: 5 μm.

Given that STX17 is implicated in the dynamics of various organelles, including the ER, Golgi and mitochondria [4,10], we examined the effects of STX17 on organelle dynamics in STX17 KO cells. In the absence of STX17, the Golgi apparatus labeled by GM130 was largely unchanged compared to those in wild-type cells (Figure 4A,B). Also, there was no obvious difference in ER morphology labeled by Calnexin or in ERGIC morphology labeled by ERGIC53 (Figure 4A,B) in STX17 KO cells or wild-type cells. One striking difference between STX17 KO and wild-type cells was in the shape of mitochondria. The length of mitochondrial tubules was significantly increased in the STX17 KO cells (Figure 4C). This observation supported a positive role of STX17 in mitochondrial fission.

### 3.4. STX17 is Crucial in Cellular Responses to Divergent Stresses

**Figure 5:**
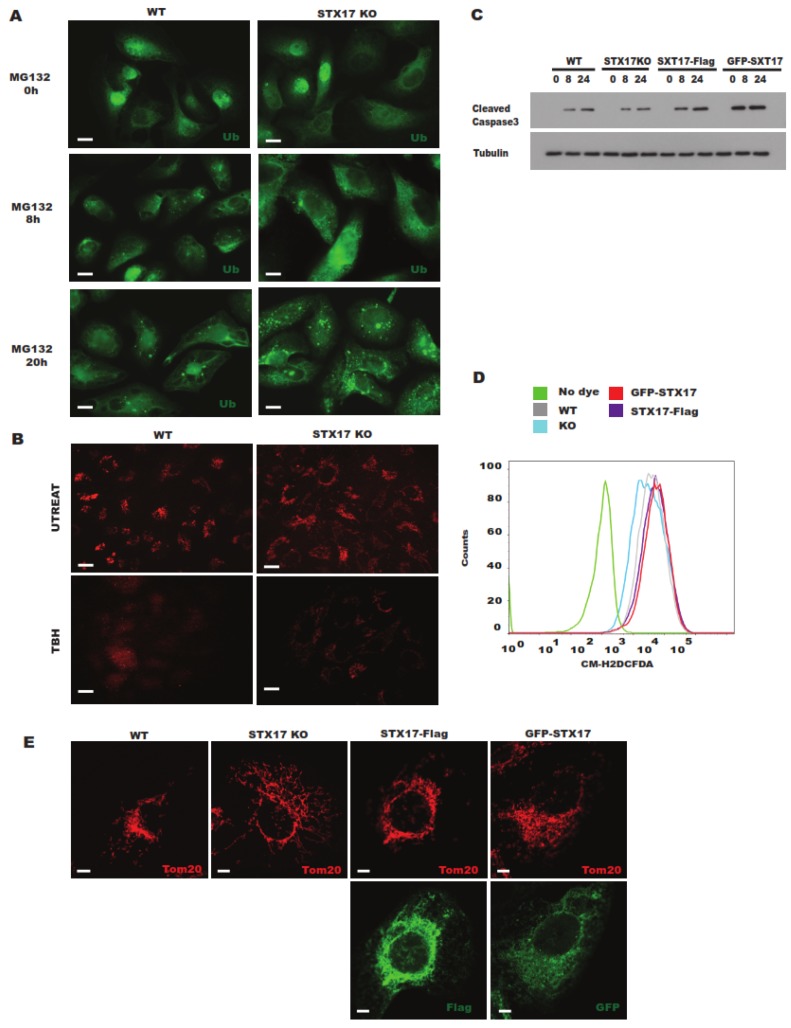
Divergent functions of STX17 in different cellular responses. (**A**) STX17 ablation led to ubiquitin aggregate accumulation. WT or STX17 KO cells were treated with MG132 for 0 h, 4 h, 8 h or 20 h. Cells were then fixed and stained with FK2 antibody. These images are representative of at least three independent experiments. (**B**) Mitochondrial membrane potential was examined in WT and STX17 KO cells using ΔΨm-dependent dye TMRE after TBH treatment. (**C**) Caspase activation of STX17 KO, WT, FLAG-STX17 and GFP-STX17 cells upon tBH treatment. The cleavage of caspase 3 was examined by immunoblotting analysis. (**D**) Measurement of total cellular ROS in CM-H2DCFDA stained WT, STX17 KO, FLAG-STX17 and GFP-STX17 cells by flow cytometry. (**E**) Flag-STX17 and GFP-STX17 expression rescued mitochondria length to a normal level. Mitochondria were marked by tom20. FLAG-STX17 and GFP-STX17 were stained using FLAG and GFP antibodies, respectively. Scale bar: 5 μm.

Protein aggregates are mainly degraded through the ubiquitin–proteasome pathway and the autophagy–lysosome pathway [16]. Proteasome inhibitor MG132 treatment blocks the ubiquitin–proteasome pathway and activates the autophagy–lysosome pathway. We examined the protein aggregate clearance ability in the absence of STX17. STX17 KO and wild-type cells were treated with MG132 for prolonged time courses and ubiquitin aggregates were stained by anti-Ub antibody. Significantly more Ub aggregates were observed in STX17 KO cells compared to in wild-type cells (Figure 5A), suggesting that STX17 knockout cells are defective in protein aggregate clearance, probably due to being incapable of autophagic degradation. 

Since mitochondrial fusion is promoted in STX17 KO cells, we wondered whether mitochondrial integrity is affected in the absence of STX17 upon oxidative stress. Cells were treated with tert-butylhydroperoxide (tBH, an ROS inducer) and the mitochondrial membrane potential was measured using the fluorescent probe TMRE (tetramethylrhodamine ethyl ester). We found that the mitochondrial membrane potential was largely protected in STX17 KO cells upon tBH treatment, whereas in controls cells, the mitochondrial membrane potential was almost completely lost (Figure 5B). This suggests that STX17 negatively regulates mitochondrial integrity. 

Given the important role of STX17 in mitochondrial fission, we examined the role of STX17 in the cellular response to oxidative stress. We anticipated that STX17 knockout would sensitize cells to oxidative stress due to the general protective role of autophagy. To our surprise, STX17 knockout cells were drastically resistant to tBH treatment, which induces robust oxidative stress and the cleavage of caspase 3 was attenuated in STX17 KO cells, supporting compromised caspase activation and apoptosis in the absence of STX17 (Figure 5C). The production of general ROS was decreased in STX17 KO cells upon tBH treatment (Figure 5D), consistent with the resistance of these cells to tBH. These results suggest that STX17 plays distinct roles in autophagy and mitochondrial dynamics. 

STX17 participates in mitochondrial dynamics by regulating mitochondrial fission machineries by serving as a Drp1 receptor [4] and by regulating autophagosome–lysosome fusion by forming an autophagic SNARE complex [5] but it is not clear which function of STX17 provides protective effects in apoptosis. In order to distinguish the roles of STX17 in autophagy and mitochondrial dynamics which contribute to apoptosis, GFP-STX17 was used for functional dissection. STX17 with a GFP tag on its N-terminal blocks autophagosome–lysosome fusion due to the sterical effect of the GFP tag [17]. Consistent with a previous report [4], STX17 knockout increased the length of mitochondria and expression of GFP-STX17 rescued the length of mitochondria to a normal level; however, autophagosome–lysosome fusion was not rescued (Figure 2C and Figure 5E). Interestingly, apoptosis was also rescued by GFP-STX17 expression and was even higher compared to in wild type cells (Figure 5C). Of note, the apoptosis was only partially rescued by the expression of STX17-Flag in STX17 KO cells in which STX17-Flag has normal function in both autophagosome fusion and mitochondrial elongation (Figure 2C and Figure 5C,E). Consistently, the increased ROS level was higher in GFP-STX17-expressing cells compared to in STX17-Flag-expressing cells or wild-type cells (Figure 5D). Taken together, these results suggest that the distinct roles of STX17 in autophagy and mitochondrial fission regulate apoptosis in opposite ways. 

## 4. Discussion

The previous reports that STX17 plays roles in both autophagosome formation and autophagosome–lysosome fusion with different phenotypes in STX17 knockdown cells are controversial [5,11]. In order to confirm the role of STX17, we generated STX17 KO cell lines in different cells and performed multiple autophagy assays to study the effects of STX17 on autophagy. Based on our results in STX17 KO cells, autophagosome formation is not altered but autophagosome–lysosome fusion is inhibited, confirming the function of STX17 in autophagosome–lysosome fusion. However, we cannot exclude the possibility that STX17 plays a regulatory role in autophagosome formation which may be caused by a different knockdown efficiency.

Interestingly, we found that autophagosome–lysosome fusion is not completely blocked in STX17 KO cells, which suggests that STX17 is not the sole SNARE involved in autophagosome–lysosome fusion. There are probably other redundant SNARE proteins in this fusion process or other SNARE proteins mislocalize to the autophagosome in the absence of STX17 and replace the function of STX17 [15]. 

STX17 is also important for mitochondrial fission, wherein its function is probably mediated by its interaction with Drp1 at the mitochondria–ER contact sites [4]. We also found mitochondrial elongation to be evident in STX17 knockout cells. The elongation of mitochondria in STX17 knockout cells provides an additional benefit to escape autophagic degradation and also likely provides protection from apoptosis [1,18]. Interestingly, the loss of STX17 had no obvious effect on ER–ERGIC–Golgi morphology. It is likely that there is some other redundant mechanism to maintain the architecture and dynamics of these organelles in the complete absence of STX17. 

**Figure 6:**
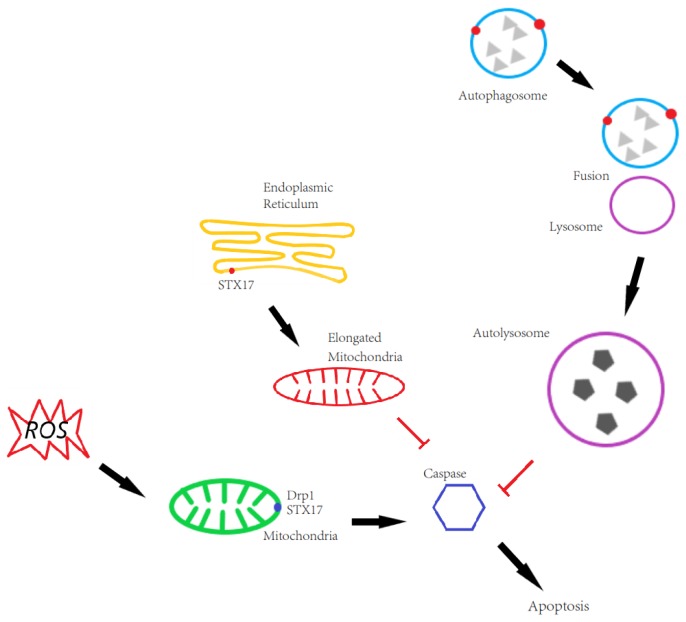
The role of STX17 in regulating apoptosis. On autophagy, STX17 KO blocks autophagosome-lysosome fusion and thus in a way caspase aggregation induces cell apoptosis. On mitochondria, STX17 KO blocks mitochondrial fission and elongated mitochondria provides an additional benefit to escape ROS induced autophagic degradation and thus spares from cell apoptosis.

STX17 is involved in both autophagy and mitochondrial dynamics. On mitochondria, STX17 promotes mitochondrial fission by regulating Drp1 localization and activity in fed conditions, whereas STX17 is transferred to MAM and autophagosomes for autophagosome biogenesis and autophagosome–lysosome fusion, respectively. This results in competing roles of STX17 in apoptosis due to its functions in autophagy and mitochondrial dynamics. Our data also show that GFP-STX17 promotes apoptosis, probably because GFP-STX17 promotes mitochondrial fission which is positively related to apoptosis. Meanwhile, GFP-STX17 blocks autophagosome–lysosome fusion, which inhibits cell death in multiple ways. It is counted as another contributing factor to apoptosis. In line with our hypothesis, Flag-STX17 expression, which not only rescued mitochondrial fission but also rescued autophagosome–lysosome fusion, decreased apoptosis compared to GFP-STX17 (Figure 6). 

Cell death includes apoptosis, necrosis and autophagic cell death. It is known ROS can induce apoptosis but not autophagic cell death, though ROS induced autophagy. Probably, ROS inhibited autophagic cell death in some way and induced the transition from autophagic cell death to apoptosis. In STX17 KO cells autophagy is deficient, which may further promote the autophagic cell death transition to apoptosis. 

## 5. Conclusions

In summary, we studied the function of STX17 in a genetic way; we not only dissected the roles of STX17 in autophagy and mitochondrial dynamics but also demonstrated STX17 in different organelles plays competing roles in ROS production and apoptosis.

## Abbreviations

SNARE: SNAP (Soluble NSF Attachment Protein) Receptor; STX17, syntaxin17; SNAP29, Synaptosomal-Associated Protein 29; VAMP8, Vesicle-associated membrane protein 8; ATG14, autophagy-related protein 14; HOPS, homotypic fusion and vacuole protein sorting; Drp1, Dynamin-related protein 1; KO, knockout; WT, wild-type; LC3, microtubule-associated protein 1 light chain 3 (MAP1LC3); ER, endoplasmic reticulum; ERGIC, the ER–Golgi intermediate compartment; CRISPR, clustered regularly interspaced short palindromic repeats; Cas9, CRISPR-associated protein 9; sgRNA, single guide RNA; RNAi, RNA interference; CQ, chloroquine; mRFP, monomer red fluorescent protein; GFP, green fluorescent protein; tBH, tert-butyl-hydroperoxide; TMRE, tetramethylrhodamine ethyl ester; ROS, reactive oxygen species.
